# Benign bone and soft tissue tumors of the foot

**DOI:** 10.1530/EOR-22-0116

**Published:** 2024-11-08

**Authors:** Carlo Biz, Andrea Angelini, Ilaria Fantoni, Mariachiara Cerchiaro, Valentina Longhi, Pietro Ruggieri

**Affiliations:** 1 Department of Orthopedics and Orthopedic Oncology, University of Padova, Padova, Italy

**Keywords:** benign tumors, bone, foot, soft tissues

## Abstract

- Tumors of the foot are a heterogeneous group of neoplasms that either affect soft tissues or bone, with a predominance being benign.
- Mistakes in the diagnosis of neoplastic conditions are common.
- A correct diagnostic approach supported by radiological and histological examination is mandatory.
- In this review, we highlight current standards in diagnosis, clinicopathological presentation, and imaging features.

## Introduction

Lesions of the foot and ankle represent approximately 3% of all bone tumors, 8% of benign soft tissue tumors, and 5% of malignant soft tissue tumors (1). They include a vast array of lesions arising from skin, adipose tissue, synovium, tendons, cartilage, bones, muscles, fibrous tissue, nerves, and blood vessels. They are most commonly non-neoplastic masses or benign neoplasms (2). Malignant lesions, although rarer, should always be included in the differential diagnosis (3). Errors in the diagnosis of neoplastic conditions are common, as symptoms are often nonspecific, including, most commonly pain and soft tissue swelling. Patients usually neglect these lesions unless they are painful or they hinder the use of normal footwear (4). Diagnostic delay and misdiagnosis frequently lead to mis-statements with poor clinical outcomes.

Excluding cutaneous lesions and nevi that are frequently observed in the foot (5, 6, 7, 8), this article reviews the clinical spectrum of the most common benign bone and soft tissue tumors of the foot, focusing on their epidemiology, clinical presentation, imaging, and pathological findings in addition to mainstays of treatment to increase awareness of the incidence, presentation, and correct approach for the most common neoplasms to help physicians assure a proper diagnostic strategy and treatment.

## Soft tissue lesions

### Ganglion cysts

Ganglion cysts are benign fluid-filled masses that can arise from joint capsules, tendon sheaths, and bursae (9), containing glassy, mucinous, clear fluid. They are most common on the dorsolateral aspect of the foot (10). They mostly affect young adults; the male-to-female ratio is 1:3. The pathophysiology of these lesions remains unclear. The most widely accepted theory relates their formation to chronic irritation and degenerative changes in connective tissue that lead to the formation of cystic spaces (11). Clinical presentation depends on the size and location of the cyst. Peripheral nerve symptoms due to ganglion cysts are not common (10). Management of symptomatic ganglion cysts ranges from observation to aspiration/injection and surgical excision (12). Asymptomatic cases are commonly managed with observation. Treatment of the ganglion is indicated once patients experience weakness, pain, and difficulty during daily activities or if the cyst has increased drastically in size (13). Although superficial, palpable cysts can be aspirated blindly in the office, ultrasound (US) guidance is important when the mass is deeper, smaller, and/or located near sensitive structures such as arteries and nerves. Traditionally, the mainstay of surgical treatment has been open ganglionectomy. Although aspiration/injection has been associated with greater rates of recurrence compared with excision, surgery often results in increased morbidity, recovery times, and costs (12).

### Tenosynovial giant cell tumors

Tenosynovial giant cell tumors (TSGCTs) are benign soft tissue tumors of the extremities, arising from the tendon sheath and periarticular soft tissues of small joints of the upper and lower extremities (14). Many terminologies have been used to define this type of tumor, including giant cell tumors of the tendon sheath, extra-articular pigmented villonodular synovitis, pigmented villonodular bursitis, and pigmented nodular synovitis of the tendon sheath (15). Although the ankle and foot constitute the next most common site after the hand and wrist, this tumor is rare when compared with upper limb lesions (16), representing 3–5% of all TSGCTs found in the body (17). Although it may present at any age, the peak incidence of TSGCTs occurs between the ages of 30 and 50 with slight female predominance (18).

**Figure 1:**
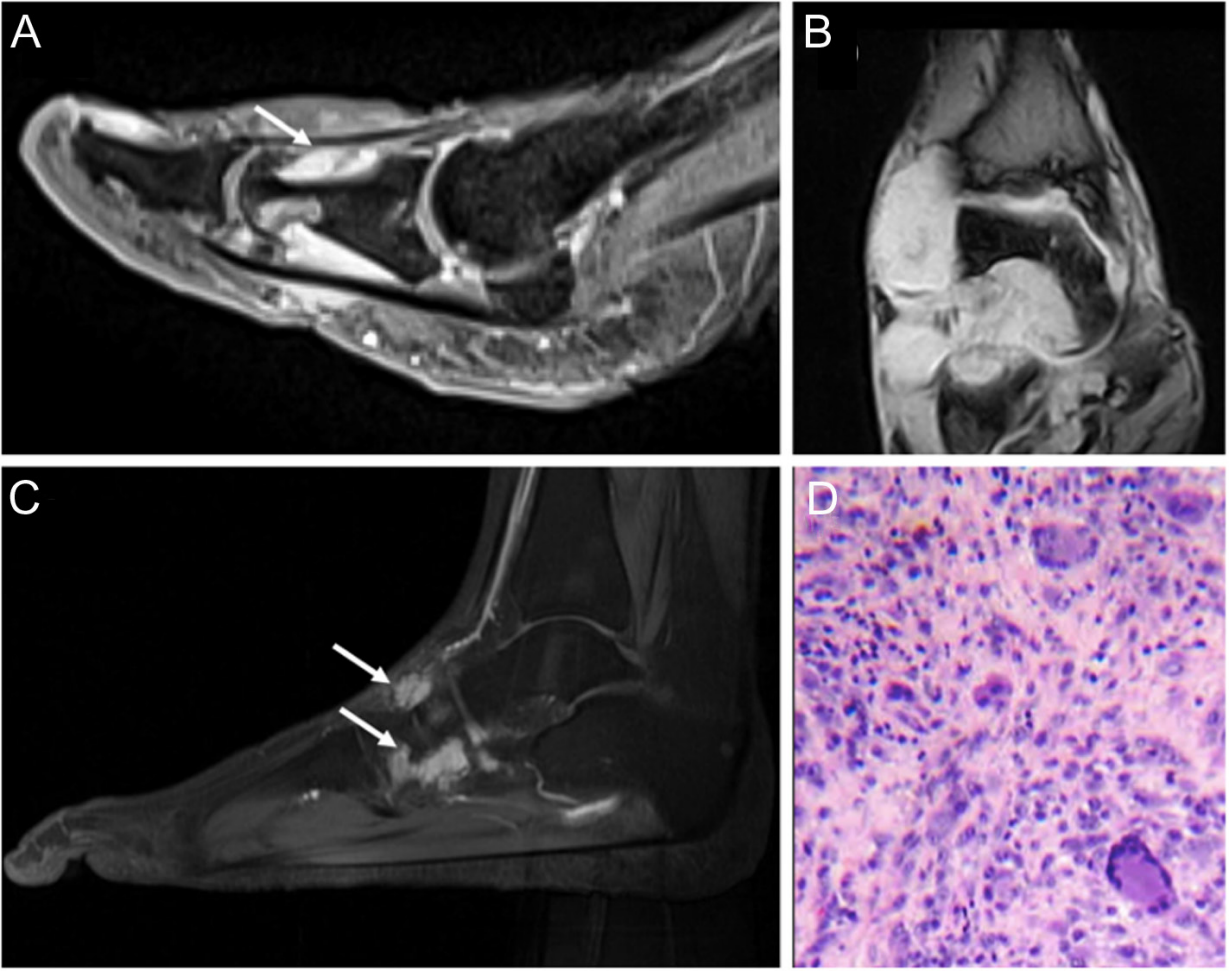
(A) Tenosynovial giant cell tumor (TSGCT) of the right flexor hallucis longus, imaging appearance with MRI in sagittal projection, showing gadolinium-enhancement due to the abundance of capillaries in the collagenous stroma (white arrow); (B) TSGCT of the ankle with aggressive behavior in a 56-year-old female; imaging appearance at MRI with extensive bone and soft tissue involvement (C) Recurrence 12 years after treatment of TSGCT of the foot in 34-year old female patient (white arrows). (D) High power view of a TSGCT, localized type: osteoclast-like giant cells, abundant mononuclear cells, and hemosiderin pigment in the top left.

The etiology of the condition is not entirely understood; however, it is thought to be a result of an inflammatory or neoplastic process. Evidence supporting the inflammatory theory includes a history of preceding trauma prior to the development of the mass. TSGCTs are classified by anatomic site (intra-articular or extra-articular) and by growth pattern (localized types or diffuse types) (19). Localized TSGCTs are more likely to occur in the forefoot and diffuse type TSGCTs in the hindfoot. Physical examination frequently reveals a non-pulsatile soft tissue mass loosely adhered to deeper structures (20). It is more commonly found on the dorsum of the foot (17). Clinically, the masses range from painless to symptomatic with great variability in size (20). Symptoms, when present, include discomfort upon weight bearing and a limitation of motion depending on the location and proximity to joints. Neurological symptoms have been observed and can be caused by compression of local nerves (17). On radiographic evaluation, a mass of increased soft tissue density is often noted with osseous changes in only 10–20% of cases. Thus, radiographs may be of little value unless osseous destruction has occurred. MRI shows a well-circumscribed lesion isointense to skeletal muscle on T1 and T2 imaging with areas of mixed signal intensity secondary to ferromagnetic hemosiderin deposition in the localized subtype (21). Gadolinium enhancement is secondary to the abundance of capillaries found in the collagenous stroma (20) (Fig. 1A, B, and C). Macroscopically, tumors range in color from dark red to brown to light brown or yellow. Diffuse tumors exhibit synovial changes characterized by a tangled mat of red-brown folds (22). On microscopic examination, the lesion is comprised of multinucleated giant cells, collagenized stroma, hemosiderin pigmentation, and lipid-laden foam cells (20) (Fig. 1D).

The current recommended treatment for TSGCTs is surgical excision; incomplete tumor removal increases the risk of recurrence (20). Treatment options are completely different considering growth pattern subtypes. In the localized type, well-circumscribed nodules affecting only a small part of the synovium usually do not require preoperative histologic confirmation (except when synovial sarcoma is suspected in the differential diagnosis) (21). Localized TSGCTs can be removed arthroscopically or with open surgery. The tumor must be dissected gently, without allowing any seeding, and the surgeon should not hesitate to remove a cuff of the tendon sheath, part of a capsule, periosteum, or even part of a tendon to ensure that all pathological tissue is removed (14). In the diffuse type, there is an infiltrative growth involving substantial parts of the synovium, especially in the ankle joint. For these cases, arthroscopic removal should be avoided since it favors further dissemination. Careful preoperative planning is important to achieve a complete synovectomy, with good oncological and functional outcomes.

The reported recurrence rate is about 20–23% in the ankle and subtalar joints. The clinical progression of osteoarthritis in patients with GCTTS of the foot and ankle is not clear; however, in cases of severe osteochondral damage, salvage procedures such as ankle arthrodesis have been described (22).

### Schwannoma

**Figure 2:**
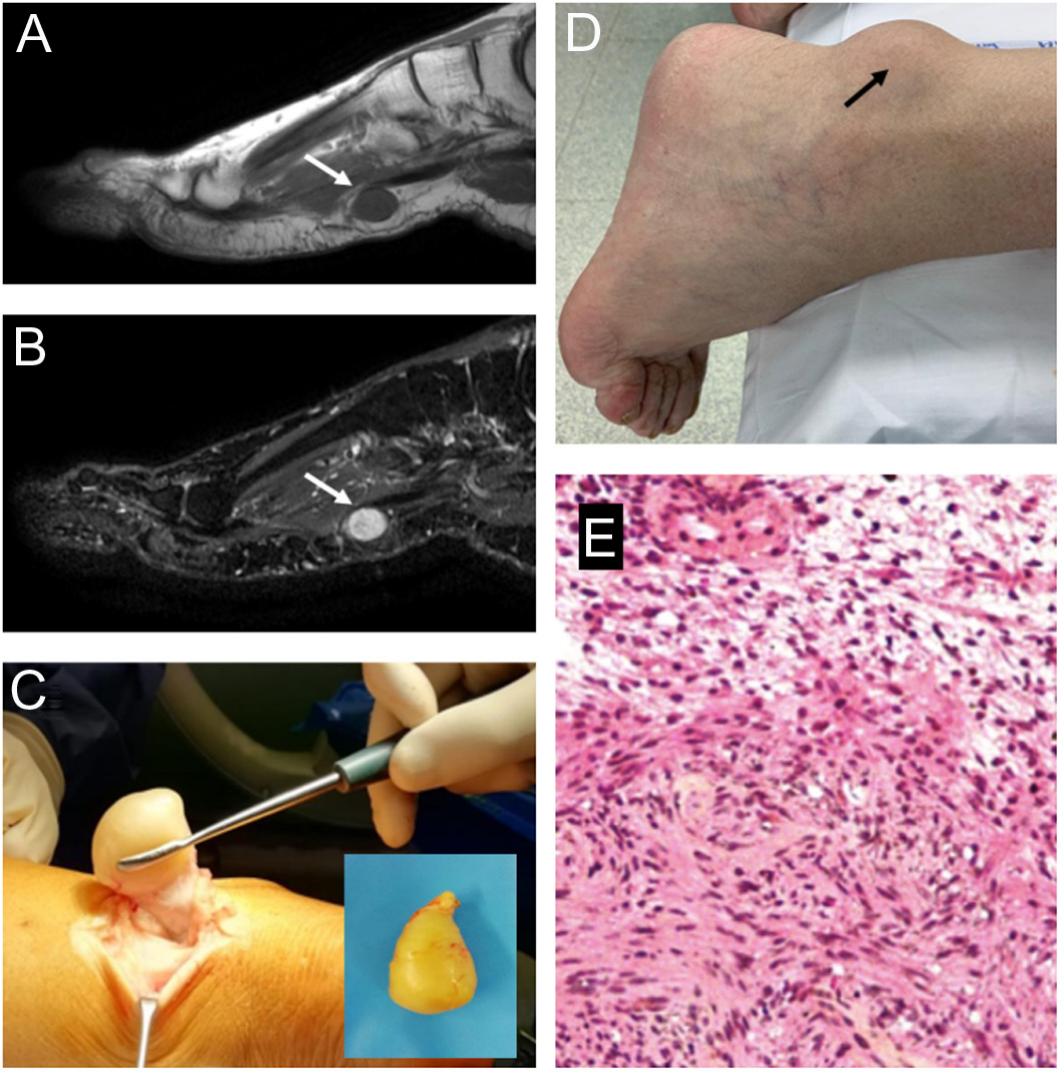
Imaging appearance of left plantar Schwannoma (white arrows), hypointense on sagittal T1-weighted sequence (A), and hyperintense on T2-weighted MRI (B). (C) Clinical appearance of schwannoma close to the Achilles’ tendon (black arrow) in a 66-year-old male patient, described as a painful, movable, and well-defined mass; (D) surgical specimen appearance and intraoperative picture of the surgical excision show the well-capsulated mass; (E) high power view of schwannoma (H&E) show more cellular areas (Antoni A, in the bottom) composed of bland cells with spindled and oval nuclei, in contrast with the loosely organized hypocellular areas (Antoni B, in the top).

Schwannoma, also called neurilemmoma (23), derives from Schwann cells located within the myelin sheath. Although it is the most common benign nerve sheath tumor in the body, it rarely affects the lower extremities, with reports ranging from 1% to 10%. The tumor does not have a gender predilection and can arise at any age, most often in the fourth decade of life (24). Ninety percent of schwannomas are sporadic. Syndromic schwannomas are seen as part of neurofibromatosis 2 (NF2) and in sporadic and familial schwannomatosis (25). Plexiform schwannoma is a rare distinctive variant accounting for 2–5% of all schwannomas (26) in which Schwann cells are organized in a plexiform pattern (27). The specific etiology of schwannomas is unknown, but prior reports suggest trauma as a potential origin (28). It usually presents as a painful, movable, well-defined mass; weakness and paresthesia can be observed when associated nerves are affected. Symptoms are mainly related to tumor location, especially in weight-bearing or easily compressed regions, including the plantar aspect and interdigital spaces. The Tinel sign can be elicited when the tibial nerve is affected (23). Radiography is usually negative, although bone remodeling due to pressure and impingement from the tumor, without signs of bone invasion, can be detected (29). US examination of schwannoma usually exhibits a round or ovoid, solid, well-delineated, hypoechoic homogeneous mass. MRI is the most valuable tool for evaluating the lesion, not only for location, size, and texture but also for relationships with nerves and other surrounding anatomical structures. Typically, schwannomas appear as isointense or hypointense to surrounding skeletal muscle on T1-weighted images and hyperintense on T2-weighted images (23) (Fig. 2A, B, and C).

Histologic presentation is highly variable. Classic features include encapsulation, a lymphoid cuff (particularly in visceral locations), clusters of hyalinized vessels, areas of increased and decreased cellularity, and nuclear palisading (25). US-guided biopsy of the lesion should be obtained when possible (30).

Asymptomatic patients with small tumors should only be monitored clinically. The reason to operate on a patient with benign neurogenic tumors should be based on the balance between the risks and benefits of the surgery. Surgical excision is necessary for large tumors arising from major peripheral nerves in the extremities and in cases of compressive symptoms. Surgery consists of the excision of the lesion after the incision of the epineurium, which allows for the sparing of the parent nerve due to its eccentric location, preserving neurological function (29) (Fig. 2D and E). Recurrence is extremely rare (less than 1%) unless the tumor tissue is incompletely excised (23). It is estimated that 1% of cases have malignant potential (30), but it is difficult to distinguish schwannomas from malignant peripheral nerve sheath tumors (29). Conventional malignant peripheral nerve sheath tumor (MPNST), epithelioid MPNST (EMPNST), and angiosarcomas have been reported to arise in conventional schwannomas (25).

### Neurofibroma

Neurofibroma is a benign tumor of the peripheral nerves, composed of Schwann cells, perineurial-like cells, fibroblasts, and transitional cells (31). Neurofibroma represents up to 5% of all benign soft tissue tumors. It tends to affect young patients, with no sex predilection. Further sub-classification of neurofibroma into localized, plexiform, and diffuse varieties is based predominantly on morphological evaluation. Localized neurofibroma accounts for 90% of these lesions (32). Solitary neurofibromas usually present as small polypoid masses, while diffuse neurofibromas have a more plaque-like appearance. Plexiform neurofibromas are large, complex tumors with a ‘bag of worms’ appearance, usually near large spinal roots. Diffuse and plexiform neurofibromas may be pigmented. Sporadic and diffuse neurofibromas only rarely progress to MPNSTs. Plexiform neurofibromas are pathognomonic of neurofibromatosis and have a significant risk of malignant transformation. Neurofibromas are composed of uniformly distributed, comma-shaped spindle cells with bland nuclei and inky chromatin in a variably myxoid to collagenous stroma (25). Plexiform neurofibromas are all congenital, typically resulting from a second genetic event in the second week of gestation (33). In contrast, dermal neurofibromas form postnatally, especially around puberty and during pregnancy, implying a hormonal role or sensitivity. The molecular pathogenesis of sporadic dermal neurofibromas is not well described (34).

These tumors can be asymptomatic while small but will present with compressive neuropathy symptoms as they become larger (35). The most challenging differential diagnosis with cutaneous neurofibroma is with spindle cells and desmoplastic melanomas (36). MRI is the investigation of choice; neurofibromas show a ‘target sign’, characterized by a low-density center and hyperintense rim of the mass on the T2-weighted images. MRI also helps with locating and determining the size of the mass and extent of nerve infiltration by the tumor, crucial to preoperative planning (35). Treatment is still controversial; however, excision is the only definitive treatment. The indications for surgical intervention include pain, dysfunction, diagnostic biopsy, and/or suspected malignancy (37).

### Lipofibromatous hamartoma

Lipofibromatous hamartoma is a rare, benign, soft-tissue neoplasm generated by the proliferation of mature adipocytes and nerve sheath fibroblasts. Usually, it develops on the volar aspects of the hands, wrists, and forearms, whereas it is rare in the lower limb (38). Patients usually present in the first three decades of life (39). Its pathogenesis is unknown. Some tumors are believed to be congenital and lack an underlying family history. Previous trauma and chronic irritation of the nerves are likely precipitating factors. The lesion presents as a soft, gray-yellow, fusiform, sausage-shaped mass. Microscopic examination typically shows fibrofatty tissue growing along the epineurium and perineurium, surrounding and infiltrating the nerve trunk (38). Its clinical symptoms usually result from the compression of affected nerves with involvement of sciatic, tibial, peroneal, calcaneal, and sural nerves. Clinically, with the slow-growing enlargement of the mass, patients typically describe pain, tenderness, numbness, and a tingling sensation in the surrounding area (40). The investigative study of choice is MRI, which shows a typical coaxial cabling sign appearance on T1-weighted series owing to low-signal nerve bundles surrounded by high-signal fibrolipomatous tissue. Management is dependent on the symptoms. Debulking surgery for aesthetic reasons can be considered if the risk to the nerve is low, and resection should be considered if the symptoms are progressive and recalcitrant (39).

### Adipose tissue tumors (lipomatous tumors)

Lipomas are benign solitary tumors composed of fat cells (41). They are the most common connective tissue tumors in adults. The peak incidence occurs between the ages of 40 and 60 and rarely occurs in children (42). Despite accounting for >16% of all soft tissue tumors in the body, soft tissue lipomas are very rare in the foot. They are thinly encapsulated and usually superficial (43). Deep masses tend to be larger and may present as fullness or asymmetry compared with the contralateral extremity. Deep lipomas can arise between or within muscle, classified as inter- or intra-muscular, respectively (42). Intramuscular lipomas are rarer, accounting for less than 1% of all lipomas. They are deeply seated (44), arising between the muscle fibers and passing through the intermuscular septa, infiltrating surrounding tissue (45). When palpable, lipomas have a soft consistency. Lipomas typically do not cause pain unless they entrap the nerves or apply pressure to adjacent tissues. The risk of malignancy is higher with older age, male sex, larger mass size, and lower extremity location (46). Pain, rapid growth, firmness, immobility, and skin changes are concerning clinical characteristics of malignancy; however, they are not consistently present, and their absence does not exclude malignancy (44).

**Figure 3:**
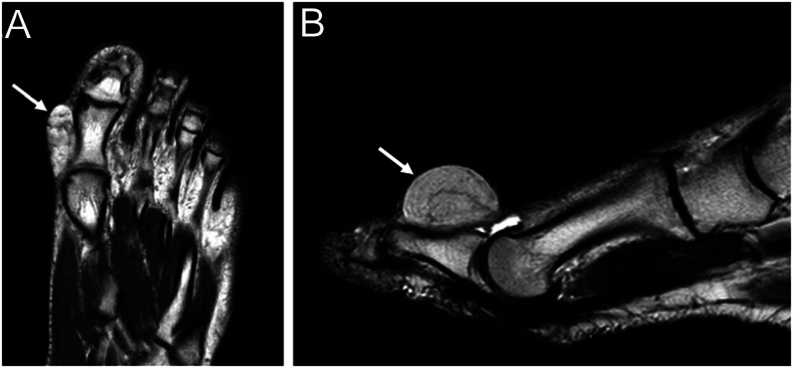
Imaging appearance of superficial left-foot lipoma of first ray (white arrows), hyperintense homogeneous signal on T1-weighted MRI in (A) transverse and (B) sagittal projection.

The differential diagnosis of a complete benign lipomatous tumor (lipoma) with other more aggressive subtypes is difficult and may be misdiagnosed by the unwary on imaging and/or pathologic evaluation (47). The WHO designates intermediate lipomatous tumors with the term atypical lipomatous tumor (ALT) used in the limbs and well-differentiated liposarcoma (WDLS) for deep lesions arising in the trunk (48). ALT are less likely to recur than WDLS even if they are similar from a histopathologic point of view (48). The amplification of the molecular marker MDM2 is useful to differentiate a simple lipoma (this feature is not present) from more aggressive tumors, through immunohistochemistry (80% sensitivity) or DNA FISH/RNA *in situ* hybridization (92% sensitivity) (49). MRI will typically show a hyperintense homogeneous signal on T1-weighted images and a hypointense signal on T2-weighted and short T1 inversion recovery images (50) (Fig. 3A and B). Both lipoma and ALT show primarily fat signal intensity on MRI, and the literature agrees on the absence of specific imaging criteria to differentiate the benign from the intermediate form. Liposarcoma should be considered whenever a heterogeneous signal is seen on short T1 inversion recovery images (43). An asymptomatic lipoma can be managed conservatively with observation. If the mass is increasing in size, cosmetically unacceptable, or symptomatic, it can be removed surgically with a marginal excision (42). Surgical excision is the best way to treat a symptomatic intramuscular, infiltrating lipoma. The recurrence rate of intramuscular lipoma is high, with reported recurrence rates of 3–62.5% (44).

### Plantar fibromatosis

Plantar fibromatosis, or Ledderhose disease, is a benign hyperproliferative disorder of the superficial plantar aponeurosis. It is frequently bilateral and multinodular, occurring in the central medial, non-weight-bearing areas of the plantar foot (51) with a reported frequency of about 1–1.75/100 000; it is rare in children, with the greatest prevalence in the sixth decade, especially affecting the male population. Although its pathogenesis is unknown, it is quite commonly associated with palmar fibromatosis (Dupuytren’s disease) and penile fibromatosis (Peyronie’s disease) (52); its cooccurrence with Dupuytren’s disease is 9–25%, while concurrence with Peyronie’s disease is 4% (51). This pathology is believed to have a hereditary component. Other contributing factors include repetitive trauma, long-term alcohol consumption, chronic liver disease, diabetes mellitus, and epilepsy (53). Its pathogenesis consists of three phases. The first is the proliferative phase, in which there is cellular proliferation with ‘no purposeful arrangement’. This stage is followed by the involutional phase, in which the alignment of fibroblasts is adjusted along the tension lines. The last phase is the residual phase, in which the cellular components are largely replaced by fibrous tissue, and the whole structure becomes acellular, often nodular and tendon-like (54).

Plantar fibromatosis is characterized by insidious onset with slow growth and a mean symptomatic period between 6 and 72 months, characterized by the presence of firm nodules about 1–2 cm situated most often on the central and medial plantar fascia. The normal aponeurosis is progressively replaced by abnormally thick collagen fibers leading to sclerosis of the entire plantar fascia. Even though it is benign, plantar fibromatosis can have aggressive local manifestations, progressively replacing the normal aponeurosis, causing pain, walking disability, problems of balance, contractures, and toe deformities. The diagnosis is clinical; it rarely requires confirmation. US shows an isoechoic nodule with a heterogeneous structure and thin hyperechoic septa with clear-cut margins and without any fluid collection or calcifications within the internal structure. It shows no vascularity. It does not invade adjacent tissues (52). MRI can be successfully used to establish disease severity. It shows well-demarcated nodules with low-intensity signal on T1-weighted sequences (areas of dense collagen) and low-to-intermediate signal intensity on T2-weighted sequences. A low signal intensity on T2 is typical of the residual phase. Subcutaneous fat necrosis, keloids, ganglion cysts, lipomas, desmoid tumors, and foreign-body reactions are the main differential diagnoses. Microscopic examination shows nodule-organized proliferation of cells with oval-shaped nuclei having small nucleoli and fine chromatin with the preponderance of type III collagen and no identifiable mitosis. The optimal treatment for this condition is unknown (54). In the early stages, conservative treatment should always be the first approach. Local injection of steroids has been shown to reduce symptoms, although it conveys the risk of dermal atrophy. High-energy shockwave therapy provides improvement of symptoms. Radiotherapy shows good outcomes, but concerns exist about side effects regarding the risk of secondary malignancies. Operative treatment is indicated for those with significant pain or neurovascular involvement, for lesions large enough to affect standing or walking, or when the diagnosis is uncertain. Local excision is associated with a high rate of recurrence, both in primary and recurrent cases. Partial fasciectomy and wide excision have the lowest recurrence rates, whereas complete plantar fasciectomy has more risk of wound healing problems and the necessity of skin grafts. Recurrent lesions are generally multiple and should be treated more aggressively (55).

### Glomus tumor

Glomus bodies are thermoregulatory cells that function as specialized modified myoarterial structures in the deep dermis, playing a role in temperature and blood flow regulation. Glomus tumors account for less than 2% of all soft tissue tumors and are categorized into solitary or multiple presentations, with solitary lesions being the most often encountered (56). They are more common in women between 30 and 50 years old (57). The most common site for these tumors is the subungual area of the fingers; however, there are reports of glomus tumors in many other locations. They are relatively rare in the foot due to the lower local concentration of glomus bodies (58).

Glomus tumors usually present as small, round, bluish nodules measuring a few millimeters in diameter visible through the nail plate. The classic presentation of a glomus tumor consists of a triad of spontaneous pain (80%), point tenderness (100%), and cold sensitivity (63%) (59). Several clinical tests can be performed for the clinical diagnosis of a glomus tumor: Love’s pin test involves the application of pinpointed pressure to the suspected area. The point at which severe pain is located identifies the area of the glomus tumor. The Hildreth test is performed by inducing transient ischemia along the arm by applying a tourniquet. A positive test is confirmed if the patient experiences a disappearance of pain in the affected area (60). Due to the low incidence of foot glomus tumors and variable clinical presentations, delayed diagnosis is frequent. In the foot, the clinical presentation has been reportedly confused with Morton’s neuroma, flexor hallucis longus tendon injury, plexiform neurofibroma, and an ingrown toenail (58). X-rays can show typical bone erosion in 30–60% of patients. Diagnostic accuracy can be improved with MRI, where a dark outlined image can be seen in T1 and a bright outlined mass can be seen in T2. This examination can be particularly useful in the early diagnosis of this type of lesion due to its small size and the diagnostic difficulties found in the initial stages. Histopathology shows glomus cells, blood vessels, and smooth muscle cells (61). Even though glomus tumors are commonly considered benign, malignancy should be considered if they are deep-seated (under muscular fascia), larger than 2 cm, or in the presence of specific histological features such as nuclear atypia, necrosis, or mitotic activity. Fatal cases with distant metastases are occasionally reported, comprising less than 1% of all glomus tumors (62). Treatment is surgical in solitary cases: complete resection, including the joint capsule in articular locations, to avoid recurrence. The outcome after surgical treatment is marked by a high recurrence rate: 1–18% depending on the series (63).

### Intramuscular hemangiomas

Hemangioma is a benign soft-tissue tumor, comprising 7–10% of all soft-tissue tumors (64). It affects about 4–5% of children, with a higher prevalence in females and Caucasians. Hemangiomas tend to go through growth and involution phases. In most children, the lesion will appear in the first few weeks of life and reach 80% of its total size by age 3–5 months (65). Most patients initially complain of pain or swelling, although superficial lesions may be manifested by prominent vascular markings or skin discoloration. Hemangiomas can occur in either the superficial (cutaneous or subcutaneous) or deep tissues (intramuscular) (66). Malignant transformation has been reported, but it is debated (64). With a course of expectant observation, many patients may experience complete involution without significant sequelae; however, the disease frequently occurs in cosmetically and functionally sensitive areas. Hemangioma has the potential for local tissue damage, ulceration, infection, bleeding, functional impact, and pain (65). The best diagnostic tool to assess hemangiomas is MRI, which is usually diagnostic. Conservative measures for persistent and symptomatic lesions, such as compression garments, are frequently recommended. A number of alternative methods, such as carbon dioxide snow, liquid nitrogen, and radiation therapy, have been used, but all have potential complications. Excision of the tumor, injection of sodium tetradecyl sulfate (sotradecol 3%), and laser therapy have also been described as treatment options for symptomatic cases. Surgical treatment can be challenging due to the vascularity of the lesions, the tendency to infiltrate muscle, and the reported high recurrence rate (66). Percutaneous sclerotherapy is potentially less invasive than open surgical excision, can be repeated if necessary, and requires interventional imaging guidance. The commonly used sclerosing agents include ethyl alcohol, ethanolamine oleate, and polidocanol (67).

### Vascular malformations

Malformations are not true neoplasms but simply developmental malformations, believed to be the result of aberrant vascular morphogenesis during the fourth to tenth weeks of intrauterine life. They are present at birth and never regress (68). Congenital vascular malformations are classified according to the modified Hamburg Classification, which subdivides them into arterial, arteriovenous, venous, capillary, lymphatic, and combined vascular malformations (69). Vascular malformations are generally subdivided into fast-flow (e.g., arterial and arteriovenous malformations) and slow-flow lesions (e.g. capillary, venous, and lymphatic malformations). Current therapeutic modalities against vascular malformations include sclerotherapy, transcatheter embolization, laser therapy, surgical resection, and radiofrequency ablation (RFA) (70).

### Pyogenic granuloma

Pyogenic granuloma or botriomycoma is a benign vascular tumor very common in daily practice; it is more typical of extremities (71). Pyogenic granulomas are commonly present in children and young adults; the mean age at onset in children is 6.7 years. It does not show a sex predilection. The lesion has been associated with chronic low-grade trauma and certain drugs, most frequently systemic retinoids, epidermal growth factor receptor inhibitors, and Indinavir. The term pyogenic granuloma is a misnomer because no infectious agent has been identified, and microscopic examination does not demonstrate a granulomatous proliferation. Histologically, pyogenic granulomas show a well-circumscribed proliferation of small capillaries grouped into lobules by fibrous bands with or without overlying ulceration (72). Typically, pyogenic granuloma presents as a red or dark red papule or nodule, usually slow-growing but at times showing rapid growth; it can easily bleed with minor trauma. Although some pyogenic granulomas may resolve spontaneously, most will require treatment. There are various treatment options, such as surgical excision, cryotherapy with liquid nitrogen, carbon dioxide laser ablation, pulsed dye laser, Nd-YAG laser, erbium-YAG laser, diode laser, electrocoagulation, sclerotherapy with monoethanolamine oleate or with sodium tetradecyl sulfate, topical 5% imiquimod cream, topical timolol 0.5% ophthalmic gel, 1% propranolol cream, systemic steroids, intralesional bleomycin injection, radiation treatment, and photodynamic therapy with 5-aminolevulinic acid. Surgical excision is considered the most effective treatment, which offers the lowest overall recurrence rates and also provides the exact diagnosis (73).

### Myositis ossificans

Myositis ossificans is a non-neoplastic, localized tumor-like lesion of new bone formation that affects muscles, ligaments, and fascia. It most commonly affects adolescents and young adults (74). About 60–75% of cases are post-traumatic; they can either follow a major trauma or result from repetitive microtraumas (75). The most agreed etiologic mechanism includes osteoblast stimulation as a consequence of bone or soft tissue damage causing the formation of new bone, dystrophic calcifications, or calcified chondroid matrix. However, in approximately 25% of cases, there is no apparent history of preceding trauma, and in some of these cases, an infectious process has been implicated as a possible cause or the initiating factor. Other possible causes include burns, neuromuscular disorders, and hemophilia. Early in the disease, the lesion is soft and painful, and within a few weeks, a firm and often painful mass develops in the affected muscles. This lesion matures over 6–12 months and eventually ossifies and becomes painless. The lesion may cause limitations in the range of movements according to its site and size (74). Histologically, myositis ossificans follows a characteristic pattern of development with the formation of distinct zonal layers. It forms in a chronological process which reflects various stages of cellular maturation. Initially, there is a central proliferating mass made up of immature fibroblastic cells mixed with inflammatory cells. Progressing outwards to the intermediate and outer zones, the cells become more condensed and calcified, eventually forming mature bone. These characteristic layers form gradually and can be identified after about 3 weeks (76). Myositis ossificans can easily mimic a variety of benign and malignant conditions. A biopsy of the central zone of the mass can contain proliferating fibrous tissue that closely resembles a malignancy (75). Diagnosis is most frequently made using a combination of clinical and radiological findings; the imaging of these lesions is time-dependent (76). Initial radiographic changes may be seen as early as 2–3 weeks after the injury, with definite bone formation evident by 2 months. CT is a useful adjunct during this maturation phase because it provides a three-dimensional view of the heterotopic bone mass. In the acute phase, MRI is useful, helping to localize the soft tissue lesion. Once bone formation occurs, MRI becomes increasingly difficult to interpret. Treatment of myositis ossificans in the acute phase consists of rest, ice, short-term splinting in muscle tension, and elevation followed by an early range of motion. Surgery often is recommended in case of limited range of motion, functional limitation, a prominent mass, or enduring pain. Surgery is performed when the bone has fully matured as judged by the presence of a cortex on radiographs, typically 6–12 months after the event; patients in the early stages of the disease process often show good results with nonoperative therapy (77).

## Bone tumors

### Intraosseous lipoma

Intraosseous lipoma is a rare benign tumor accounting for less than 1 per 1000 of all primary bone tumors that arise within the bone marrow. It is composed of mature adipose (fat) tissue and is usually asymptomatic and incidentally detected on imaging, although pathological fractures have also been observed in extremely rare cases (78). When it specifically affects the foot, intraosseous lipoma is usually observed in the calcaneus (32% of all lower limb bones) (79). When evaluating a case of intraosseous lipoma of the calcaneus, several differential diagnoses should be considered based on similar radiologic findings. However, if the lesion is present in both heel bones, this represents a pathognomonic finding (80). The exact cause of intraosseous lipomas is not well understood, but most of the literature supports that they develop from the proliferation of adipose tissue within the bone marrow. Other theories suggest that trauma, chronic irritation, or genetic factors may play a role in their formation (81).

**Figure 4:**
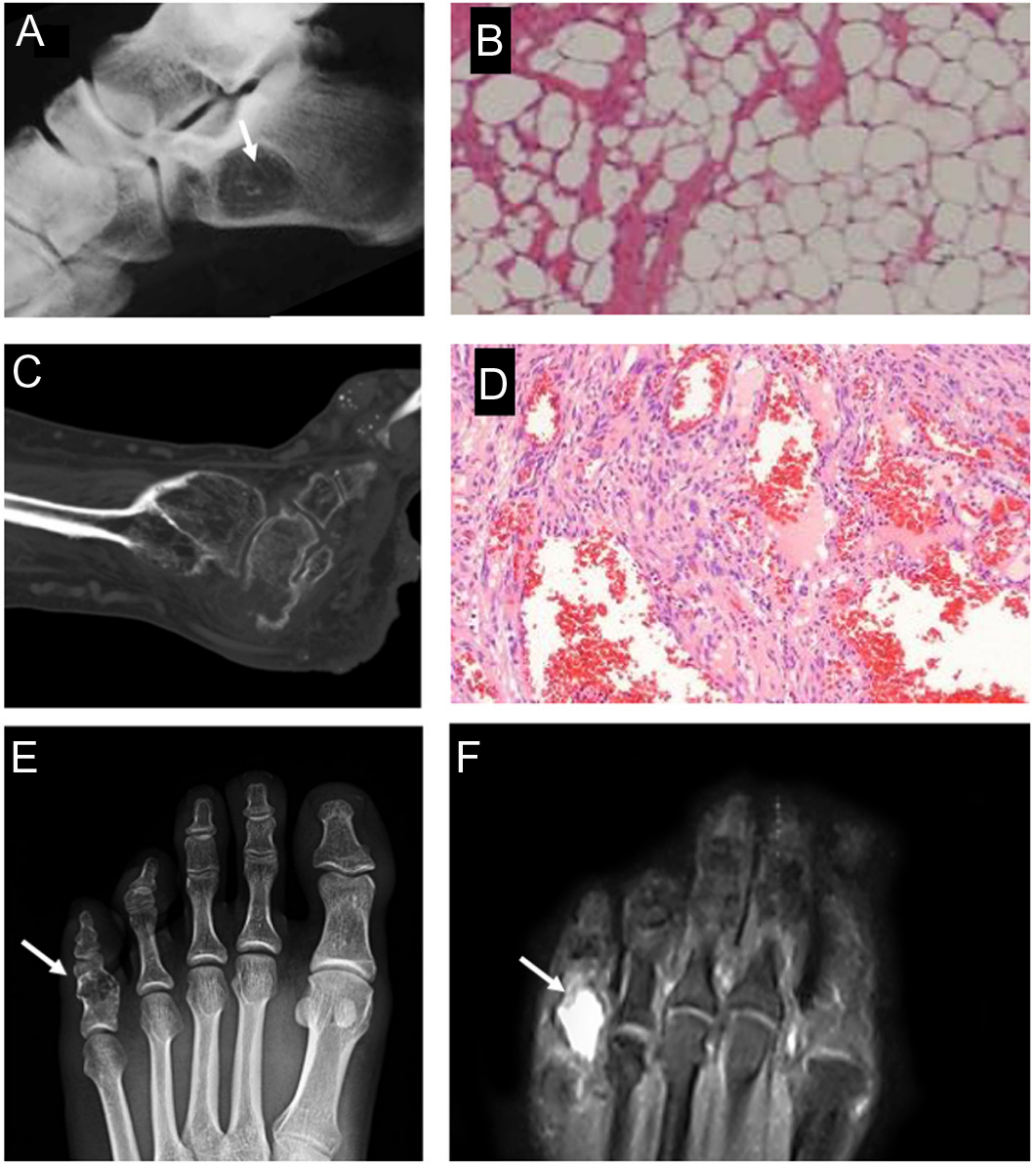
(A) Intraosseous lipoma of the calcaneus with the typical central mineralization (white arrow). (B) Histology shows the typical lobules of white adipocytes. (C) Sagittal CT scan of a foot in a patient with Maffucci syndrome. (D) Microscopic appearance of a Maffucci syndrome, with numerous hemangiomas and hypocellular areas with an abundance of hyaline cartilage matrix. (E) Pathological fracture of the proximal foot phalange of the fifth finger in a patient with enchondroma (white arrows), that appears as an osteolytic lesion containing typical ‘pop-corn’ opacities on X-ray; (F) MRI shows cartilaginous tissue with a high signal intensity on T2-weighted sequence.

The diagnosis of intraosseous lipoma is typically confirmed through imaging studies such as X-rays, CT scans, or MRI (82). Radiographs may reveal a radiolucent (dark) intramedullary lesion with well-defined sclerotic borders (Fig. 4A). A CT scan demonstrates an intraosseous lesion of fat density that may have central calcification and cortical expansion, especially in later stages. MRI is particularly useful in differentiating lipomas from other bone lesions and providing detailed information about the extent and characteristics of the tumor: in early stages, intraosseous lipoma demonstrates high signal intensity in T1 and T2-weighted images and suppression on the STIR sequence. Microscopic examination shows lobules of mature-appearing adipocytes (Fig. 4B).

Treatment for intraosseous lipoma of the foot depends on several factors, including the size, location, symptoms, and potential complications (83). Asymptomatic or small lipomas that do not cause any functional impairment may be managed conservatively with regular monitoring and imaging to ensure stability. If the lipoma is large, symptomatic, or causing structural damage to the bone, surgical intervention may be considered in terms of curettage with or without bone grafting of the affected bone (83).

### Enchondroma

Enchondroma is the second most common benign primary hyaline cartilage-forming bone tumor, accounting for 12–24% of primary bone tumors. It arises in bones that form by endochondral ossification. These tumors typically present during the third to fourth decade of life and are most frequently located within the distal appendicular skeleton (84). Half of these lesions are found in the hands and feet. They are typically found within the medullary cavity of the bone. The most common location of an enchondroma is in the metaphyseal region. It is composed of mature hyaline cartilage (85). The most common locations for enchondroma of the foot are the phalanges and metatarsal bones. They can arise as solitary lesions or as multiple lesions, such as in the context of Ollier disease or Maffucci syndrome (86). Whereas Ollier disease is a rare sporadic non-hereditary skeletal disorder in which enchondromas develop near the growth plate cartilage, Maffucci syndrome is a rare genetic disorder characterized by the presence of multiple enchondromas and hemangiomas (Fig. 4C and D). It is usually diagnosed in childhood or early adolescence and can affect various bones and soft tissues throughout the body, causing deformities and functional impairment, including in the foot (86). The hemangiomas can cause swelling, discoloration, and sometimes pain, and may be present at birth or develop later in life (84, 86).

The majority of enchondromas are asymptomatic and discovered incidentally; however, when symptomatic, they usually present with pain and swelling of varying durations. Symptoms are usually related to larger lesions with cortical thinning, endosteal scalloping, malignant transformation, or pathologic fractures (87). In long bones, a common differential diagnosis includes bone infarct and low-grade chondrosarcoma. X-ray findings include typical chondroid matrix mineralization in stipples or arcs and rings (Fig. 4E). There should be no endosteal scalloping, no cortical breaks, no periosteal reaction or soft tissue mass. With MRI, the lesion shows low signal changes on T1 and high signal changes on T2-weighted sequences with a lobular growth pattern (88) (Fig. 4F). Enchondromas are composed of lobular, relatively cell-poor hyaline cartilage, often separated by a zone of reactive bone formation (encasement). Chondrocytes have nuclei with condensed chromatin and are evenly dispersed. Binucleated cells are rarely seen, and mitosis is absent. Degenerative features, such as ischemic necrosis or calcifications, can be prominent. Enchondromas in the phalanges display more myxoid features, and the lesions may have increased cellularity (89). Atypical cartilaginous tumors (ACT), in contrast, demonstrate destructive permeation into the surrounding host bone trabeculae and bone cortex. Differential diagnosis between the two entities is mainly based on imaging, clinical, and histologic criteria.

For small and asymptomatic lesions, simple observation and careful follow-up are suggested. Surgery is indicated for symptomatic lesions as well as for larger lesions (greater than 3–4 cm) even if these lesions are asymptomatic. Surgical treatment includes complete excision without grafting, chemical cauterization, intralesional curettage and bone grafting, subtotal resection, and grafting, or total resection (90). In the surgical scenario, the standard treatment is curettage of the bone with augmentation of the vacant space. The decision to consider resection as a treatment option depends on several factors, including the characteristics of the enchondromas (if there is evidence of rapid growth, enlargement of the lesion, or suspicious features for malignant degeneration), the symptoms experienced (pain, discomfort that affects daily activities, inefficacy of pain medications or orthotic devices), and the impact on foot function (deformities, joint stiffness, limitations in foot and ankle movement).

### Osteoid osteoma

**Figure 5:**
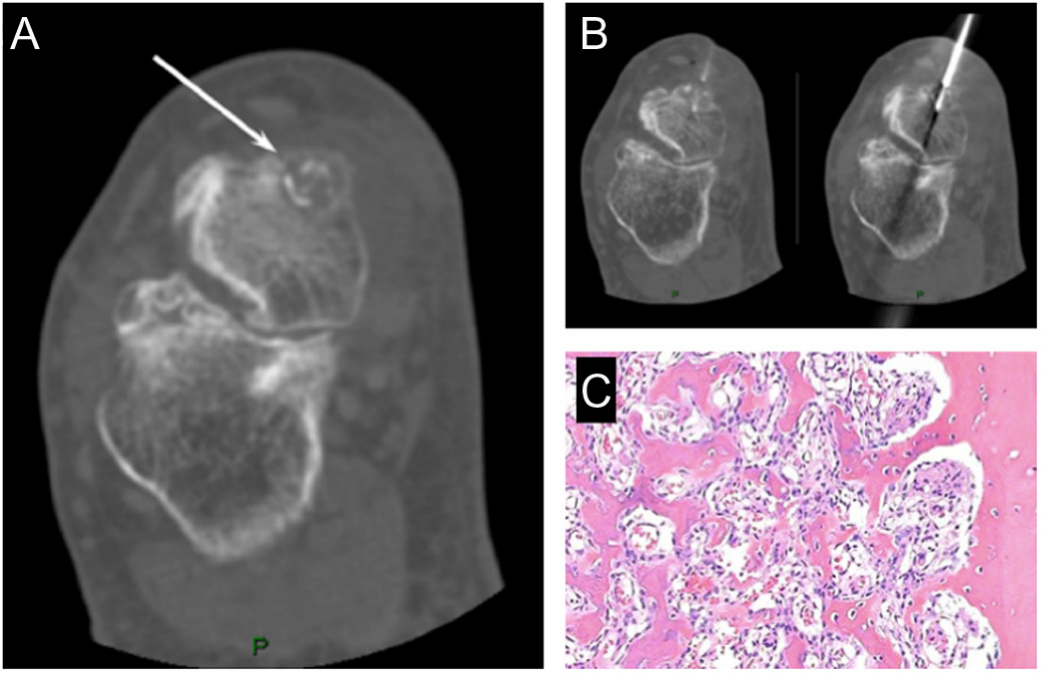
Osteoid osteoma of the talus in a 45-year-old male. (A) CT scan clearly shows the nidus (white arrow); (B) CT scan during different phases of the thermoablation. (C) Histology shows trabeculae of woven bone of variable thickness, fibrovascular stroma, a well-defined border of the lesion and a dense sclerotic reactive border.

Osteoid osteoma is a benign tumor that originates from osteoblasts. Approximately 50% of osteoid osteomas occur in long bones of the lower limbs. The incidence in phalanges of the foot is reported to be 1% (91). It generally occurs in the second and third decades of life (75% of the cases are in patients younger than 25), and men outnumber women at a ratio of 2 to 1 (92). About 2–10% of cases occur in the talus, which is the fourth most commonly affected bone; the other bones of the foot are less frequently involved (93). Osteoid osteoma is composed of osteoid and woven bone and consists of a centrally located radiolucent nidus, usually 1–10 mm in diameter, typically surrounded by a variable amount of sclerotic reaction (Fig. 5). Histologically, the nidus is composed of highly vascular tissue, rich interwoven osteoid trabeculae and mineralized matrix with a fibrovascular stroma.

The classic presentation is local swelling and intense dull pain that worsens at night. Because of the elevated level of prostaglandins in the nidus, which causes pain and vasodilatation, symptoms are relieved dramatically with the use of salicylates or other nonsteroidal anti-inflammatory drugs (94). On X-rays, these lesions are typically <1.5 cm in size and appear as small, radiolucent nidi surrounded by reactive rims of sclerotic bone. CT is the best modality to assess and diagnose these lesions, especially in the foot and ankle region due to the small, complex anatomy; it is reported to have a sensitivity of 96.4% (Fig. 5A). MRI has been reported to miss the diagnosis in about a third of cases, and it usually shows extensive bone edema, which makes it difficult to see the nidus of the lesion (87).

The standard treatment traditionally has been surgical excision, but it has significant disadvantages: difficulties in identifying the nidus during surgery and reaching the lesion in deep locations (hip joint), higher aggressiveness, and higher risk of postoperative complications (95). Percutaneous thermal destruction of the vascular-rich nidus is the current treatment of choice and can be performed using a laser or RFA with a success rate of >90% (Fig. 5B). However, in selected cases, the proximity to the chondral surface or neurovascular structures should change the preference to an open technique. The recurrence rates with both open and percutaneous techniques range from 0% to 15%, with similarly successful results (96).

### Osteochondroma

**Figure 6:**
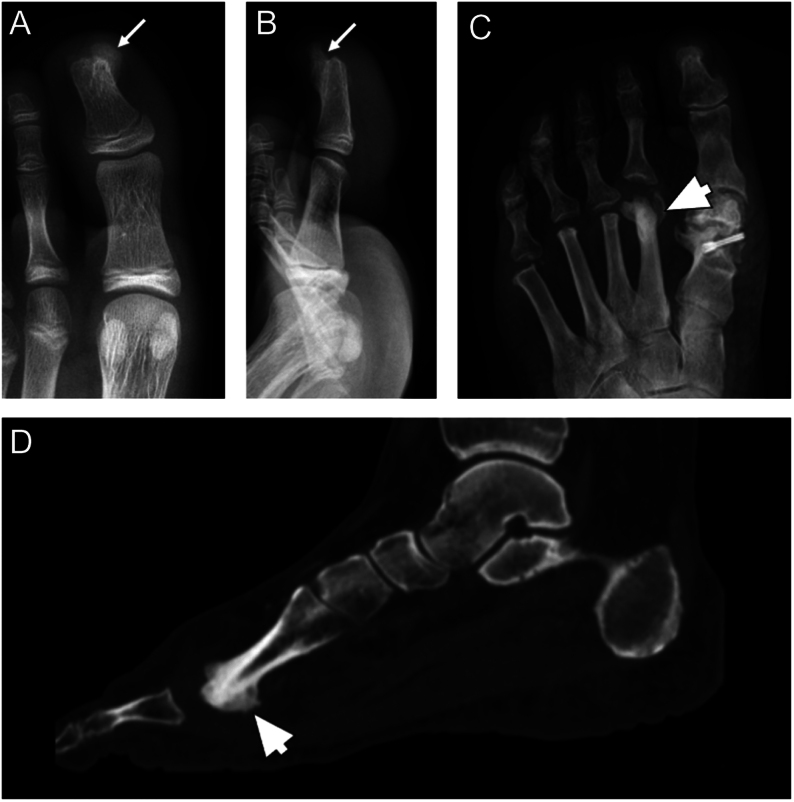
Antero-posterior (A) and lateral (B) radiographs of osteochondroma of the distal foot phalange (white arrows), showing a pedunculated bony mass with continuous growth of medulla and cortex; (C) Nora’s lesion of the second metatarsal bone (arrow’s head) showing mineralizing exophytic outgrowth with a characteristic lack of medullary involvement on antero-posterior radiograph and (D) sagittal CT scan.

Osteochondroma, or solitary osteocartilaginous exostosis, is the most common of all bone tumors, accounting for about 50% of all benign skeletal tumors. An osteochondroma is a developmental growth defect, pathogenically related to a focal herniation of the lateral component of the epiphyseal plate (97). Osteochondromas can be solitary or multiple. Multiple tumors are associated with a syndrome termed hereditary multiple exostoses. Solitary tumors constitute 20–50% of all benign bony tumors. The male/female ratio is 2.5:1; most tumors are found during rapid skeletal growth (98). Histopathologically, an osteochondroma contains bone marrow that continues directly into the medullary cavity of the underlying bone (99). An osteochondroma usually presents as a painless excrescence localized to the metaphysis of a long bone, symptomatic for local mechanical irritation. Vascular complications such as arterial thrombosis, arteriovenous fistula formation, pseudoaneurysm, claudication, acute ischemia, and arterial rupture can also develop if the degree of friction and pressure is sufficient (100). Most osteochondromas can be diagnosed directly using regular plain radiographs (Fig. 5A and B). The typical image manifestation can be a sessile or pedunculated bony mass on the bone surface. CT images can also show whether both the medulla and cortex have grown continuously with the stalk of the lesion. MRI allows measurement of the cartilage cap thickness, assessment of malignant transformation, and evaluation of its relationship with other structures (Fig. 6A and B). Solitary osteochondroma has been believed to have a very low risk of malignant transformation. Changes in symptoms, particularly new-onset pain in a previously painless lesion and acceleration of its speed of growth, should always be considered as a warning for possible malignant transformation. The cartilage cap thickness is a valuable indication of the potential for malignant transformation. Malignancy should be suspected if the thickness is >2 cm in adults and 3 cm in children. Erosion or destruction of adjacent bones should also be considered as a sign of secondary chondrosarcoma (101). A suspected osteochondroma that shows warning signs of malignancy should be surgically excised and examined histologically. Other indications for surgical removal include pain, shoe-fit problems, skin breakdown, and neurovascular compromise. Surgical excision is the mainstay of treatment for pedal osteochondromas resulting in shoe pressure and irritation. Similarly, the risk of fracture of the tumor stalk with ambulation is another indication for removal. All perichondrium should be removed during excision, as recurrence is common if any portion of the cartilage cap remains (102).

### Bizarre parosteal osteochondromatous proliferation (BPOP, Nora’s lesion)

Bizarre parosteal osteochondromatous proliferation (also known as Nora’s lesion) is a rare disorder involving the feet and hands. Typical sites include the proximal phalanx and metacarpal and metatarsal bones. Hands are four times more likely than feet to be affected. The frequency with which it affects men and women is equal. It most commonly occurs between the ages of 20 and 30 years (103). Histologically, it is characterized by differing amounts of cartilage, bone, and spindle cell proliferation as well as an unusual form of calcified chondroid tissue (104). The atypical cartilage presents marked proliferative activity resembling chondrosarcoma, disorganized ossification, plenty of irregularly calcified osteoid with benign osteocytes, and actively proliferating but benign fibrous tissue (105). Whether the whole picture can be explained by trauma or represents a true neoplastic process is questionable, as many patients do not remember an antecedent trauma. The lesion is characterized by an exophytic outgrowth. Differential diagnosis includes parosteal osteosarcoma, periosteal chondrosarcoma, osteochondroma, and subungual exostosis (106). Radiographically, BPOP presents as a mineralizing exophytic lesion consisting of bone, cartilage, and fibrous tissue. The lesion arises from cortical bone with or without osteolysis, cortical flaring, or periosteal reaction (98). A lack of medullary involvement is characteristic of BPOP (107, 108) (Fig. 6C). Nora’s lesion typically presents as a painful, bony swelling that progressively increases in size over a period of weeks to months (109). A CT scan helps to distinguish it from osteochondromas by showing the absence of continuity between the cortex and medullary cavity of the bone and the lesion (Fig. 6D). Excision is the recommended treatment for symptomatic BPOP. Wide excision can be curative, but there is a likelihood of local recurrence when intralesional excision is attempted. The rate of initial recurrence is 51%, while the rate of second recurrence is 22%. Most recurrences occur within 2 years of excision. Resection of the capsule of the lesion and decortication of the underlying cortical bone has been recommended as a means of reducing the rate of recurrence (110).

### Subungual intraosseous epidermoid cyst

Intraosseous epidermoid cysts (IECs) are squamous epithelial-lined benign cysts within the bone. They can occur anywhere throughout the body, even though these benign cystic lesions caused by the proliferation of epidermal tissue are commonly observed within the subungual region. This type of cyst is rare and typically affects the distal phalanx of the finger or toe. The pathogenesis is not clear, but certainly, IECs originate from the epidermis or by entrapment of epidermal cells within the bone and other tissues of the digits (111). In fact, they can occur due to various factors, such as trauma, inflammation, or developmental abnormalities. Subungual intraosseous epidermoid cysts usually present as a painless swelling or bump beneath the nail plate (112). They can cause nail deformities, such as nail plate elevation or splitting, and sometimes lead to chronic nail bed inflammation or infection (111, 112). The overlying nail may appear discolored, thickened, or have an irregular shape (111). To diagnose a subungual IEC at imaging exams, the following characteristics should be confirmed: at US, a round/oval anechoic or hypoechoic subungual mass is observed with lateral shadows and contains variable echogenic foci, without internal vascularity at color Doppler imaging (113). X-rays may reveal a radiolucent (dark) lesion in the distal phalanx, often adjacent to the nail bed. MRI can provide more detailed information about the extent and characteristics of the cyst, helping to differentiate it from other subungual lesions (114).

Observation should be considered if the cyst is small and asymptomatic, tracking any changes in size or symptoms with regular monitoring using imaging studies. Treatment options include surgical removal for symptomatic or enlarging cysts. The procedure involves removing the cyst along with a portion of the affected bone. It is important to ensure that the cyst and any associated nail deformities are adequately addressed during the surgery. In some cases, it may be possible to preserve the nail plate during the surgical excision. This technique aims to maintain the integrity and aesthetics of the nail while removing the cyst.

### Chondroblastoma

Chondroblastoma (CB) is a rare benign tumor that accounts for 1% of all primary bone tumors. It may affect every bone, and patients are most commonly in their second decade of life. In adults, it more commonly involves the flat and short tubular bones of the foot, especially the talus and calcaneus (115). Reported male/female ratio ranges from 2:1 to 5:1 (116).

Most patients with CB present with pain, which is often quite severe, and limitation of movement of the adjacent joint. X-rays demonstrate a destructive lesion of the epiphyseal site, often with some expansion of the bone and a sclerotic rim. Within the cartilaginous mass that contains the tumor, there is often punctate calcification (117). CT can provide valuable information in the diagnosis and evaluation of the extent of the disease; it is extremely helpful in the case of joint involvement, cortical manifestations, or any evidence of pathological fractures. CB may be associated with a secondary aneurysmal bone cyst (ABC). Microscopic examination reveals typical polygonal-shaped chondroblasts and osteoclast-like giant cells. The chondroblasts are large and closely packed with a central, basophilic, characteristically grooved nucleus and translucent cytoplasm. Scattered throughout are small, nucleated giant cells and islands of more mature cartilage. Calcifications are present in approximately 60% of lesions (118).

Although CB is a benign tumor, it sometimes exhibits an aggressive tendency to invade joints and adjacent bone. Clinically, it should be distinguished from giant cell tumors, cartilaginous mucinous fibroma, and osteosarcoma; biopsy is required for an accurate diagnosis in most cases. In general, the treatment of CB is surgery with extensive bone curettage, followed by bone grafting. In rare cases, phenol, liquefied nitrogen, and high-frequency electrode catheter application have been used as adjunctive therapy in an effort to reduce the risk of tumor recurrence. CB has been reported to recur in 10–43% of cases (119).

### Unicameral bone cyst

Unicameral bone cysts (UBCs) are benign fluid-filled cystic lesions that occur most commonly in the proximal femur and humerus. They are rare in the foot and ankle region, where they usually affect the calcaneus (87). They are typically found in skeletally immature patients with a male-to-female ratio of approximately 3 to 1 (120). These lesions are not neoplastic. Presumably, they represent a local disorder of development and bone growth (121). Most calcaneal bone cysts are asymptomatic and are identified as incidental findings after minor trauma, whereas long bone cysts are often diagnosed secondary to pain caused by mechanical weakness or pathological fractures. Pathological fracture as a consequence of a calcaneal bone cyst is reported to be rare (110), but complete healing without supplemental fixation or biologic enhancement is reported in only 14.8% (122). On X-rays, the lesion forms a more or less triangular area pointing upwards, devoid of trabecular structure, well delineated, and sometimes surrounded by a sclerotic margin, usually more visible with computed tomography. The defect may display signs of moderate chronic expansion, most often predominating inferolaterally, with cortical thinning and bowing and no periosteal reaction (123). Observation with about 3 years of follow-up has been considered the correct approach in most calcaneal UBCs that are asymptomatic and discovered incidentally on radiographs. The indication for treatment of a unicameral bone cyst is to manage symptoms – primarily pain – and to prevent a pathologic fracture (124). Unicameral bone cysts located in the feet can be managed with debridement of the cyst lining followed by bone marrow aspirate concentrate incorporation, injection of a calcium sulfate/calcium phosphate composite, injection with allogenic bone graft, and less frequently, by filling with cortisone. Surgical long bone unicameral bone cyst management includes stabilization with an intramedullary nail or cyst decompression with cannulated screws (125). Open curettage with autograft bone augmentation is the most effective procedure in terms of pain control (124), with complete relief in about 70% of cases of heel pain and cyst healing in 93% of the cases at radiographic examination (122).

### Aneurysmal bone cyst

ABCs are locally aggressive, benign, and expansile bone lesions that rarely occur in the foot and ankle region, with a reported rate between 5% and 9%. The metatarsals and the calcaneus are the most commonly involved bones (87). Nearly 70% of affected patients are aged 5–20 years, and approximately half of these cysts occur in the second decade of life. No sex predilection is reported. These lesions arise de novo in 65% of cases, especially in children and adolescents. They can, however, be secondary to a pre-existing condition. Lesions preceding ABCs include giant cell tumors, osteoblastomas, and most commonly calcaneal CBs (126, 127). Standard radiographs reveal an expansive, lytic, and proliferative lesion. CT and MRI scans may be helpful in the diagnosis of ABCs since T2-weighted MRI could detect a deformity in the involved metatarsal bone as a segmented, expansile, multiseptated lesion with a large quantity of fluid (128). The fluid levels seen on MRI are characteristic. Histopathological confirmation of an ABC is desirable before deciding on treatment, as occasionally highly malignant tumors, like telangiectatic osteosarcoma, may mimic an ABC radiologically (129).

**Figure 7:**
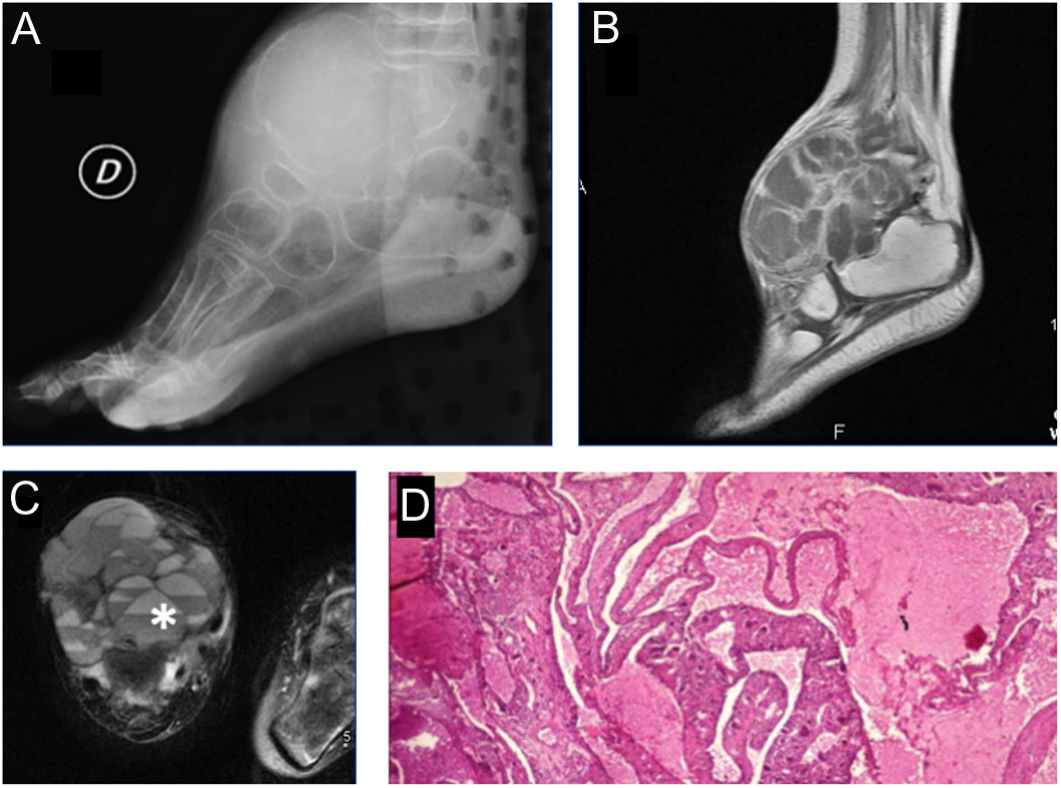
Aneurysmal bone cyst (stage 3) in an 18-year-old male. (A) X-ray appearance showing a large lytic lesion involving the proximal and middle foot. (B) Sagittal T1-weighted MRI shows deformity in the involved bone with a multiseptated lesion; (C) MRI T2-STIR in axial projection shows the typical fluid-fluid levels (white asterisk). (D) Histological view of the cellular septa containing fibroblasts, giant cells, and woven bone. There are typical cavernous spaces filled with blood, lacking endothelial lining.

Curettage with or without bone grafting is the most common treatment. The recurrence rate has been reported to be around 30%; most recurrences are thought to occur in younger patients, in major limb bones, or in patients with larger cysts. Additional physical adjuvants, such as cryotherapy, phenol, hypertonic saline, merthiolate, and polymethylmethacrylate (PMMA) cement, have been suggested to prevent recurrence (130) (Fig. 7A, B, C, D, and E).

### Giant cell tumor of bone

**Figure 8:**
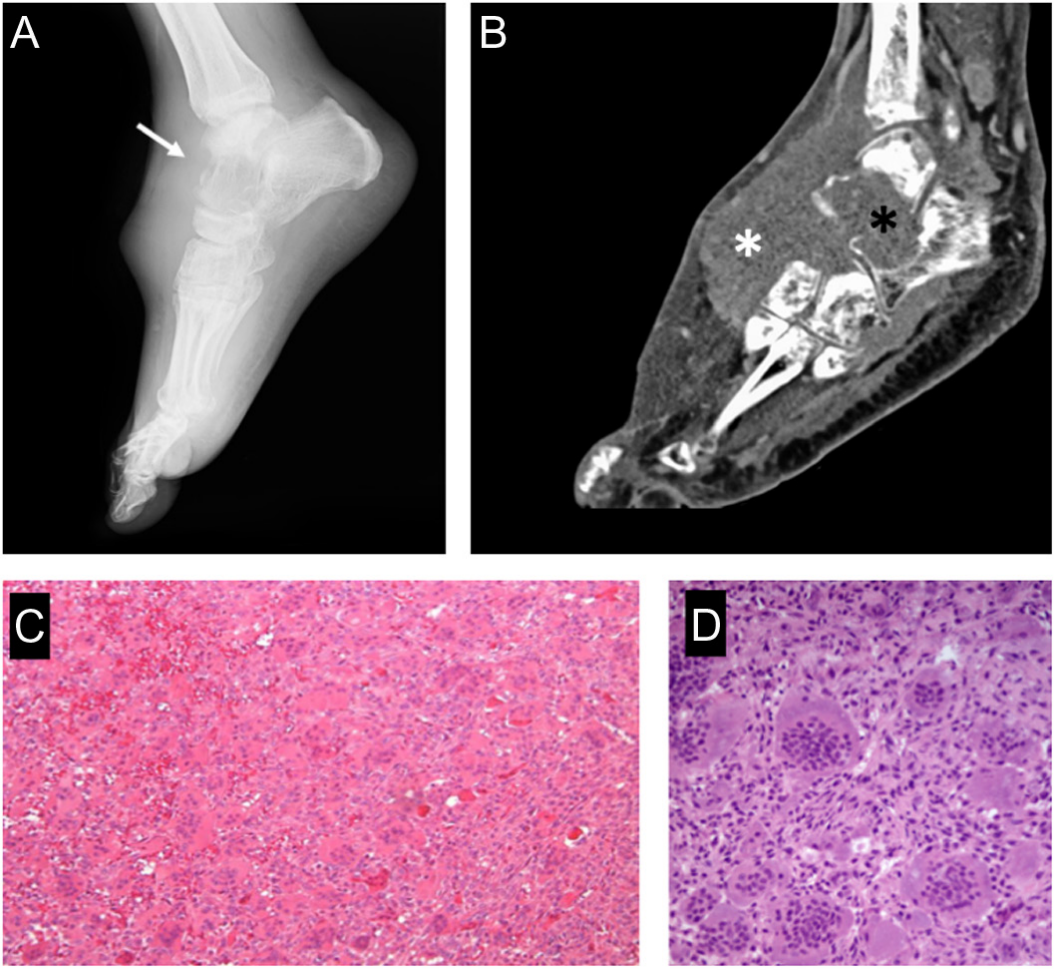
Giant cell tumor of the bone (stage 3). (A) x-ray appearance showing a characteristic purely lytic lesion, destroying the cortex (white arrow) and bulging in the soft tissues; (B) sagittal CT scan shows the aggressive pattern in bone (black asterisk) and soft tissue (white asterisk). (C) Typical histologic features with numerous osteoclast-like giant cells and mononuclear neoplastic cells. (D) high-power histologic appearance.

Giant cell tumor (GCT) is a benign, locally aggressive bone tumor rich in osteoclast-like giant cells. It represents around 5–6% of all primary bone neoplasms and 20% of benign bone neoplasms (87). Fewer than 5% of GCTs involve tubular bones of the hands and feet. It occurs most commonly in the third to fifth decades of life and affects both males and females, with a slight female preponderance. It usually presents as a solitary lesion involving the meta-epiphyseal region of long bones. Multicentricity of GCTs is extremely uncommon (131). The most common clinical presentation includes pain, swelling, and altered gait (132). Radiographically, the lesions are purely lytic and eccentrically located in the epiphyses of long bones. They usually affect the subchondral bone with frequent cortical expansion or interruption; however, intra-articular extension is rare because the subchondral bone usually remains intact (133) (Fig. 8A and B).

Histopathologically, these tumors are composed of mononuclear cells, macrophages, and uniformly distributed multinuclear giant cells. GCT is regarded as a predominantly osteoclastogenic stromal tumor. It has been shown that the giant cells in GCT are reactive osteoclasts (134) (Fig. 8C). The RANK pathway is often reported to be involved in the pathogenesis of giant cell tumors of bone. Dysregulation of the RANK ligand (RANKL)-RANK osteoprotegerin signaling cascade induces an imbalance between bone formation and bone resorption, which leads to changes in bone mass and increases osteoclast-mediated bone destruction (135). Differential diagnosis includes ABC, brown tumors (hyperparathyroidism), non-ossifying fibroma, benign osteoblastoma, and giant cell reparative granuloma (136). The standard treatment of lesions in the long bones is curettage, often with local adjuvants such as phenol, liquid nitrogen (cryosurgery), and/orPMMA to reduce the recurrence rate, which has been reported from 12% to 34%. More aggressive lesions of the long bones with soft-tissue extension, pathological fracture, or involvement of joints may be treated by en bloc resection (137).

Administration of preoperative Denosumab (a monoclonal antibody targeted against RANKL) shows clinical, radiological, and histopathological therapeutic benefits. It is associated with the formation of a calcified rim, condensed osseous matrix, and thickened cortical bone. On average, patients are generally treated for about 3–6 months preoperatively. Long-term pre-treatment with Denosumab should be avoided (because of its dose-dependent toxicity profile), and it should be limited to the minimum needed to conduct operable salvage surgery. Abrupt cessation of Denosumab will result in tumor relapse. Hence, preoperative Denosumab therapy should be continued until definitive surgical treatment becomes feasible (138).

## ICMJE Conflict of Interest Statement

RP reports that he is a consultant for Stryker and Exactech. The other authors declare that there are no relationships/conditions/circumstances that present a potential conflict of interest with the present manuscript.

## Funding Statement

There was no external funding source in support of this study. Open access was funded by the Department of Surgery, Oncology, and Gastroenterology (DISCOG) of the University of Padova.

## Ethical approval

All procedures performed in studies involving human participants were in accordance with the ethical standards of the institutional and/or national research committee and with the 1964 Helsinki Declaration and its later amendments or comparable ethical standards.
